# Bilateral Femoral Head Osteochondritis Dissecans in an Adolescent: A Report of a Rare Case

**DOI:** 10.7759/cureus.87585

**Published:** 2025-07-09

**Authors:** Alaa Al-Taie, Omar Abunima, Syed Alam, Renan Ibrahem Adam

**Affiliations:** 1 Radiology, Hamad General Hospital, Doha, QAT; 2 Radiology, Hamad Medical Corporation, Doha, QAT; 3 College of Medicine, Qatar University, Doha, QAT; 4 Musculoskeletal Radiology, Hamad Medical Corporation, Doha, QAT

**Keywords:** adolescent hip pain, bilateral hip lesions, femoral head, hip disorders, hip osteochondritis dissecans, mri hip, musculoskeletal radiology, ocd hip, plain radiography, subchondral bone lesion

## Abstract

Osteochondritis dissecans (OCD) is an uncommon condition involving the subchondral bone and overlying cartilage, typically affecting the knee. Its occurrence in the hip is rare, and bilateral involvement of the femoral heads is considered exceedingly uncommon, especially in the absence of trauma or repetitive stress. We present the case of an 18-year-old female with bilateral anterior hip pain, mainly when transitioning from standing to sitting. She reported no history of trauma, athletic activity, or systemic symptoms. Physical examination revealed normal gait and full range of hip motion without any pain. Laboratory investigations were within normal range. Anteroposterior pelvic radiographs showed bilateral subchondral lucencies in the femoral heads. MRI confirmed stable OCD lesions centered at the fovea capitis, without signs of articular surface disruption, fluid clefts, or fragment instability. Given the imaging features and the patient’s preserved function, conservative management was recommended. This case highlights the importance of considering hip OCD in adolescents with vague hip pain and emphasizes the diagnostic value of plain radiography in early detection and MRI in further characterization. Early recognition can help in preventing disease progression and reduce the risk of long-term complications such as osteoarthritis.

## Introduction

Osteochondritis dissecans (OCD) is a joint disorder characterized by focal disruption of the subchondral bone and its overlying cartilage, potentially leading to instability, fragmentation, or detachment [1]. While it most commonly affects the knee, other joints such as the elbow, ankle, and hip may also be involved [2,3]. OCD of the femoral head is a particularly rare presentation, with bilateral involvement being exceedingly uncommon, especially in adolescents [3,4]. The condition usually presents with vague or nonspecific symptoms such as hip or groin pain and may be undiagnosed without appropriate imaging. Radiographic evaluation typically reveals subchondral lucencies or irregularities, but early or subtle cases can be missed [1,5]. MRI is valuable in confirming diagnoses, assessing lesion stability, and guiding treatment decisions [5,6]. Herein, we report a rare case of bilateral femoral head OCD in an 18-year-old female, with an emphasis on radiographic and MRI findings that contributed to early diagnosis and conservative management.

## Case presentation

An 18-year-old female presented with bilateral anterior hip pain, primarily when transitioning to sitting after prolonged standing or walking. She denied trauma or high-impact sports participation. Examination revealed a normal gait and full, pain-free hip range of motion. Lab tests were normal.

**Figure 1:**
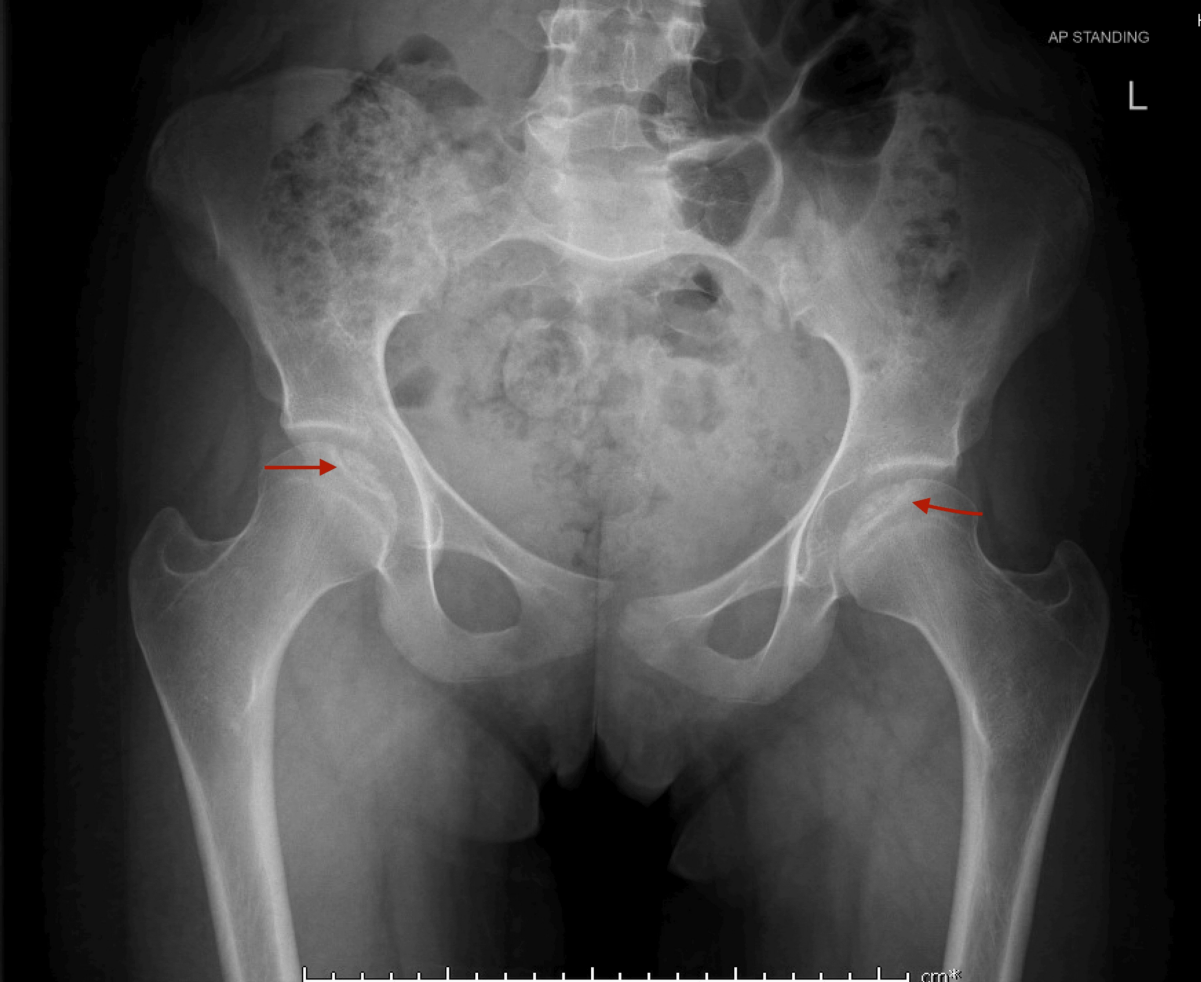
Anteroposterior pelvic radiograph showing bilateral subchondral lucencies in the femoral heads (red arrows), with no evidence of articular surface collapse or fragment displacement

Pelvic radiograph (AP view) demonstrates subtle, bilateral subchondral radiolucencies involving the anterosuperior aspects of both femoral heads. No evidence of articular surface collapse, fragmentation, or sclerosis is observed. The joint spaces are preserved, and there are no associated osteophytes, periarticular calcifications, or signs of avascular necrosis (Figure 1).

**Figure 2:**
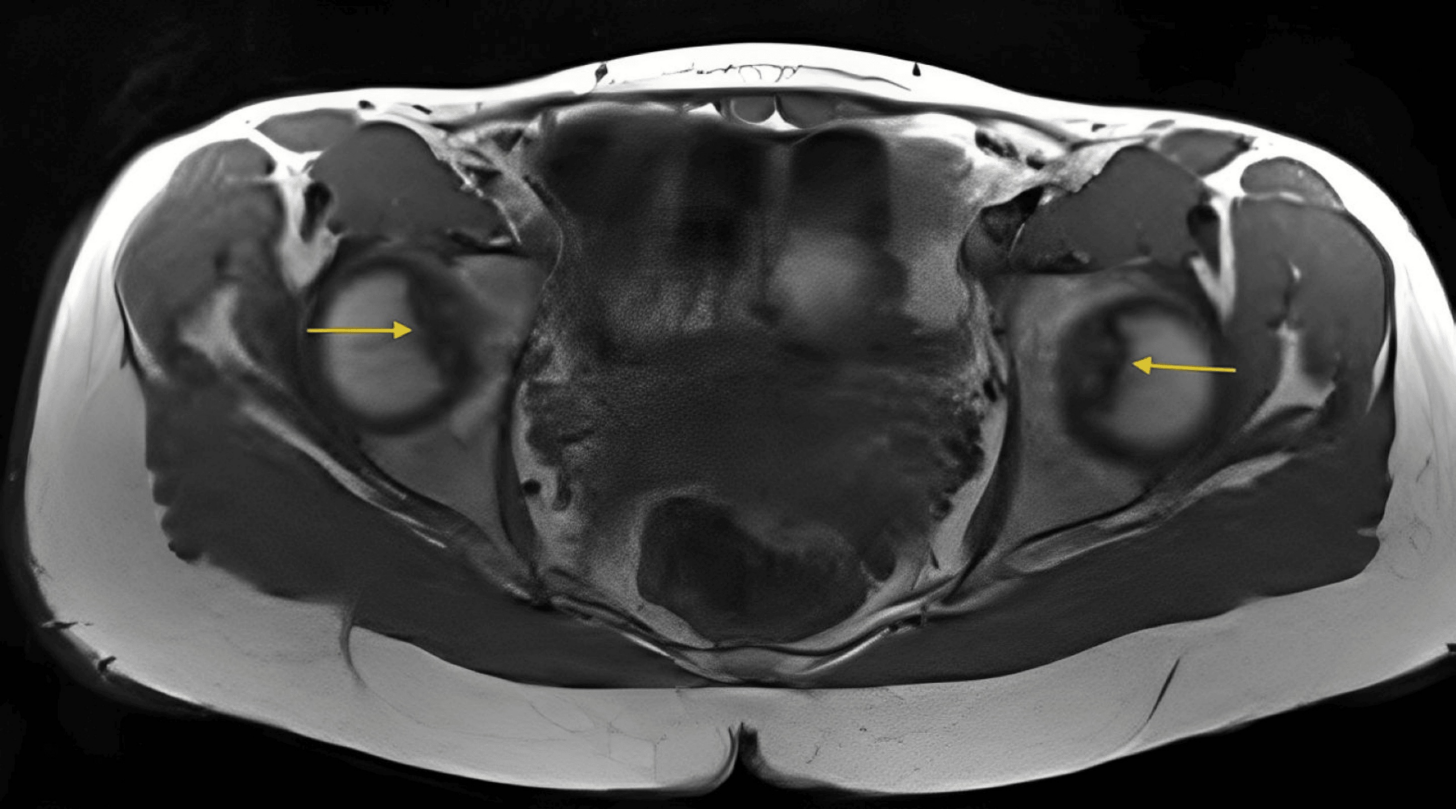
Axial T1-weighted MRI of the pelvis and hips demonstrating bilateral femoral head subchondral lesions at the fovea capitis (yellow arrows), with intact cartilage and no signs of instability

**Figure 3:**
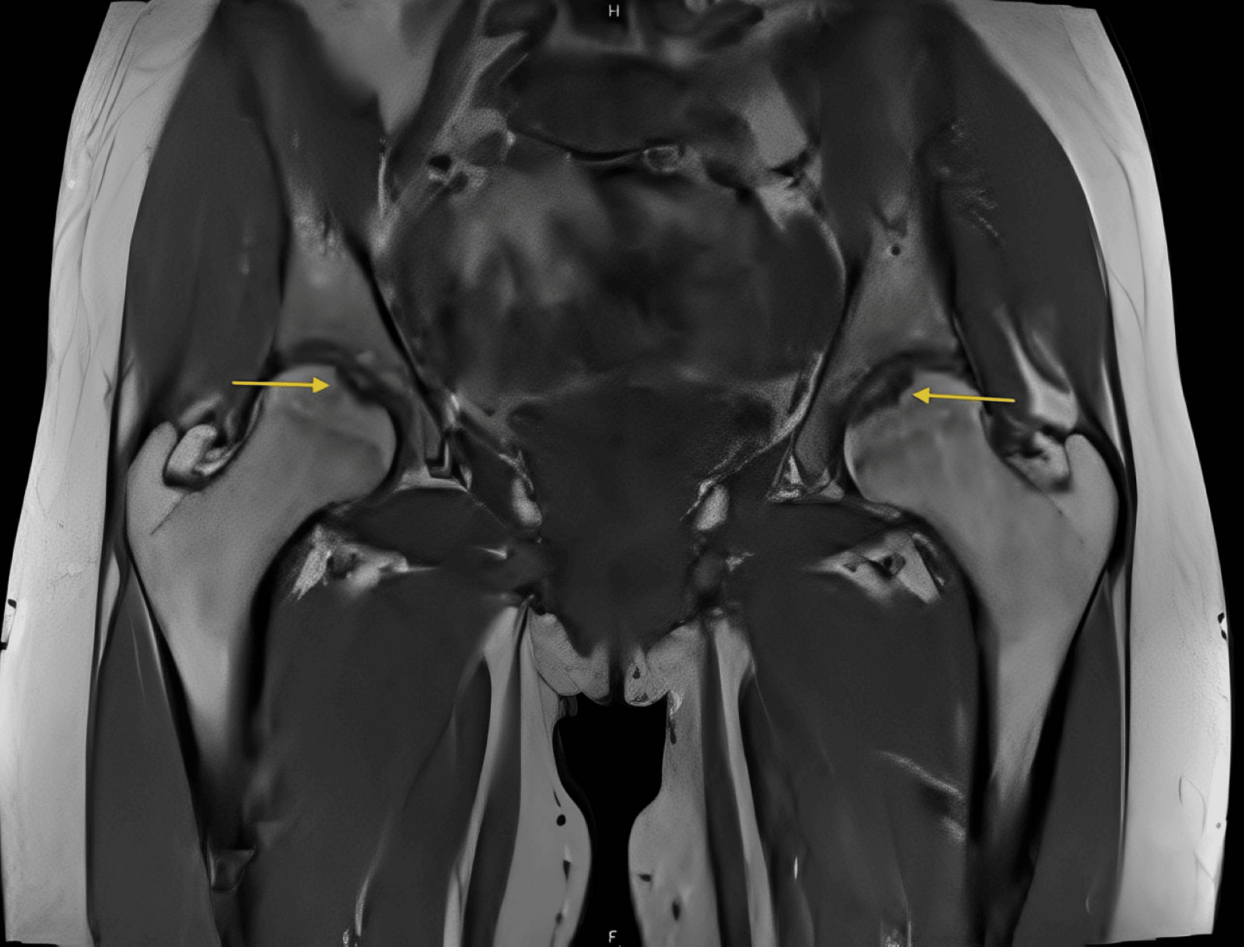
Coronal T1-weighted MRI of the pelvis and hips showing bilateral femoral head subchondral lesions at the fovea capitis (yellow arrows), with intact cartilage and no signs of instability

**Figure 4:**
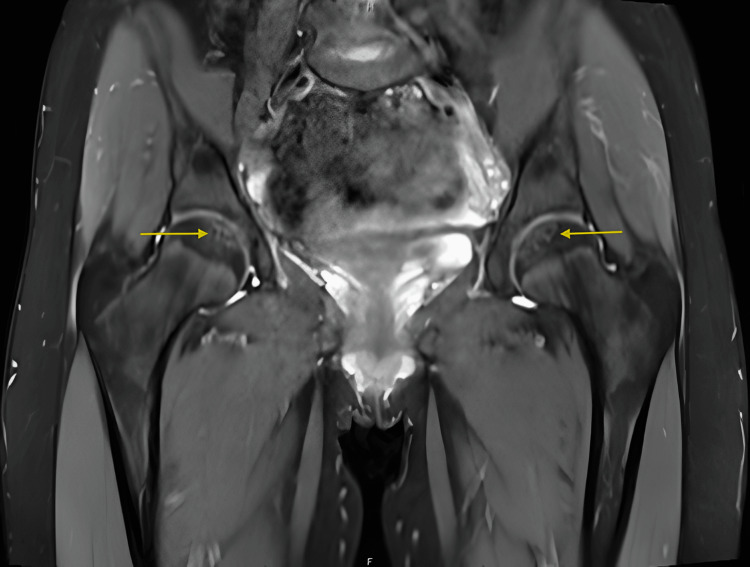
Coronal proton-density fat-suppressed MRI of the pelvis and hips demonstrating bilateral femoral head subchondral lesions at the fovea capitis (yellow arrows), with intact cartilage and no signs of instability

MRI revealed bilateral femoral heads demonstrating small subchondral T1 hypointense lesions at the fovea region with minimal surrounding T2 hyperintensity indicating mild marrow edema, intact overlying cartilage, and no evidence of fluid cleft, cystic changes, or fragment instability findings consistent with mild, stable bilateral OCD corresponding to subtle subchondral lucencies seen on radiographs (Figure 2, Figure 3, Figure 4).

## Discussion

OCD of the hip is a rare condition, particularly in adolescents, with bilateral involvement being exceedingly uncommon [2,3]. Diagnosis is often delayed due to nonspecific symptoms such as vague hip or groin pain and a lack of clinical suspicion. OCD of the hip is rare compared to the knee, ankle, or elbow, and bilateral involvement (as in this 18-year-old female) is particularly uncommon [1,2]. Plain radiographs often show lucencies but may miss cartilaginous or early subchondral changes. MRI is superior for lesion sizing, cartilage assessment, cystic change detection, and edema evaluation [3,4].

Other conditions, such as avascular necrosis, femoroacetabular impingement, and Legg-Calvé-Perthes disease, as discussed by de Angeli et al., must be considered in the differential diagnosis [6]. Hernandez et al. described pediatric cases presenting subtly [4], whereas Weaver et al. emphasized athletic adolescent hip OCD [2]. Lee et al. showed bilateral cases linked to athletic microtrauma [5]. Kim and Wenger underscored that hip OCD can cause pain in otherwise healthy children [7]. Majewski et al. described hip OCD as a diagnostic challenge requiring high-resolution, fat-suppressed MRI sequences to detect subtle lesions [8].

While advanced imaging modalities like MRI have become standard in musculoskeletal diagnostics, the importance of conventional radiography should not be underestimated. In fact, Bobin’s retrospective study on OCD of the hip in adolescents emphasized that early radiographs were sufficient to confirm the diagnosis in most cases, highlighting their diagnostic value in initial evaluation [9]. This supports the role of radiographs as a frontline tool in assessing suspected hip OCD, particularly in typical locations like the anterosuperior femoral head or, as in our case, the fovea capitis. Although MRI is often employed to evaluate lesion characteristics such as marrow edema or cartilage involvement, Bobin argued that it should serve a supplementary role rather than a primary diagnostic tool [9]. Our case mirrors this perspective, as radiographic findings alone prompted the diagnostic pathway, with MRI primarily used for confirmation and lesion stability assessment.

The absence of trauma or high-impact activity in our patient suggests a possible idiopathic or developmental etiology [7]. Conservative management is generally favored for stable lesions, especially when there is no evidence of fragmentation, instability, or articular surface disruption [4,8]. By recognizing the diagnostic power of conventional radiography, particularly in early and stable lesions, clinicians can improve diagnostic accuracy without over-relying on MRI. Greater awareness of bilateral hip OCD presentations will help guide timely intervention and prevent long-term complications such as osteoarthritis.

## Conclusions

Bilateral femoral head OCD is a rare but important differential diagnosis in adolescents presenting with unexplained hip pain. This case highlights the diagnostic value of plain radiography in early detection, even in the absence of trauma or athletic overuse. While MRI can provide additional detail regarding lesion stability and surrounding structures, conventional radiographs remain a reliable frontline tool. Increased clinical awareness of such uncommon presentations can aid in timely diagnosis, reduce the risk of long-term joint complications, and support effective conservative management in stable lesions.

## References

[1] Durur-Subasi I, Durur-Karakaya A, Yildirim OS (2015). Osteochondral lesions of major joints. Eurasian J Med.

[2] Weaver CJ, Major NM, Garrett WE, Urbaniak JE (2002). Femoral head osteochondral lesions in painful hips of athletes: MR imaging findings. AJR Am J Roentgenol.

[3] Edmonds EW, Heyworth BE (2014). Osteochondritis dissecans of the shoulder and hip. Clin Sports Med.

[4] Hernandez SG, McQueen RG, Erickson JB (2023). Femoral head osteochondritis dissecans in a child. BMJ Case Rep.

[5] Lee JE, Ryu KN, Park JS (2014). Osteochondral lesion of the bilateral femoral heads in a young athletic patient. Korean J Radiol.

[6] de Angeli LR, Serafim BL, Cordeiro FG, Bessa FS, Maranho DA (2024). Osteochondritis dissecans of the hip in Legg-Calvé-Perthes disease: case report and review. Acta Ortop Bras.

[7] Kim HK, Wenger DR (2015). Osteochondritis dissecans of the femoral head: a rare cause of hip pain in children. J Pediatr Orthop B.

[8] Majewski J, Stoeckle U, Raeder C (2019). Osteochondritis dissecans of the femoral head: a diagnostic challenge. Orthop Rev.

[9] Bobin N (2005). Osteochondritis dissecans of the hip in children and adolescents.

